# *MIR448* antagomir reduces arrhythmic risk after myocardial infarction by upregulating the cardiac sodium channel

**DOI:** 10.1172/jci.insight.140759

**Published:** 2020-12-03

**Authors:** Gyeoung-Jin Kang, An Xie, Hong Liu, Samuel C. Dudley

**Affiliations:** Lillehei Heart Institute, University of Minnesota, Minneapolis, Minnesota, USA.

**Keywords:** Cardiology, Arrhythmias, Sodium channels, hypoxia

## Abstract

Cardiac ischemia is associated with arrhythmias; however, effective therapies are currently limited. The cardiac voltage-gated sodium channel α subunit (*SCN5A*), encoding the Na_v_1.5 current, plays a key role in the cardiac electrical conduction and arrhythmic risk. Here, we show that hypoxia reduces Na_v_1.5 through effects on a miR, miR-448. miR-448 expression is increased in ischemic cardiomyopathy. miR-448 has a conserved binding site in 3′-UTR of *SCN5A*. miR-448 binding to this site suppressed *SCN5A* expression and sodium currents. Hypoxia-induced HIF-1α and NF-κB were major transcriptional regulators for *MIR448*. Moreover, hypoxia relieved *MIR448* transcriptional suppression by RE1 silencing transcription factor. Therefore, miR-448 inhibition reduced arrhythmic risk after myocardial infarction. Here, we show that ischemia drove miR-448 expression, reduced Na_v_1.5 current, and increased arrhythmic risk. Arrhythmic risk was improved by preventing Na_v_1.5 downregulation, suggesting a new approach to antiarrhythmic therapy.

## Abstract 2

Ischemic induction of miR-448 negatively regulates the cardiac sodium channel Nav1.5, and inhibiting miR-448 raises Nav1.5 and reduces arrhythmic risk after myocardial infarction in mice.

## Graphical abstract

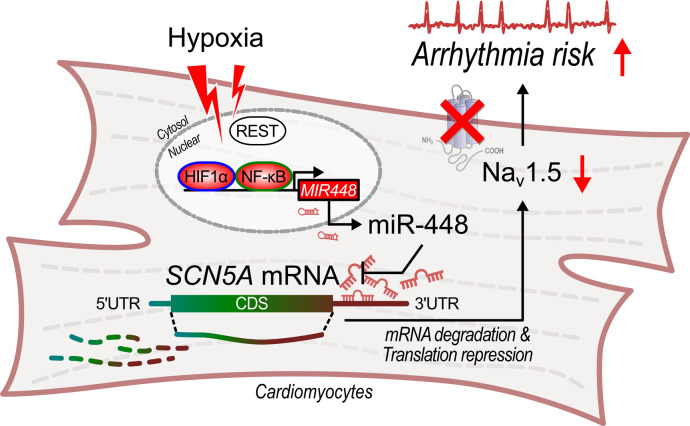

## Introduction

Cardiac ischemia is associated with arrhythmic risk; however, effective therapies are limited. The cardiac voltage-gated sodium channel α subunit (*SCN5A*), encoding the Na_v_1.5 current, plays a key role in the cardiac electrical conduction. Ischemic cardiomyopathy is associated with reduced Na_v_1.5, contributing to arrhythmic risk (1–4).

Previous studies show that the regulation of Na_v_1.5 expression depends on equilibrium between different mechanisms, such as gene transcription, RNA processing, posttranscriptional regulation by miRNA or RNA-binding proteins, protein synthesis, assembly, and posttranslational modification and trafficking (5–8). Cardiac sodium channel downregulation can be mediated by transcriptional regulation, posttranscriptional mRNA splicing, modulation of mRNA stability, translational regulation, and posttranslational modification (9–14). However, the mechanisms whereby ischemia causes Na_v_1.5 downregulation remain unclear. miRNAs are small noncoding RNA molecules (20~22 nt) that function in RNA silencing and posttranscriptional regulation of gene expression (15, 16). Here, we show that miR-448 is upregulated in cardiac ischemia and contributes to the downregulation of Na_v_1.5.

## Results

### miR-448 was upregulated with cardiac ischemia.

**Figure 1:**
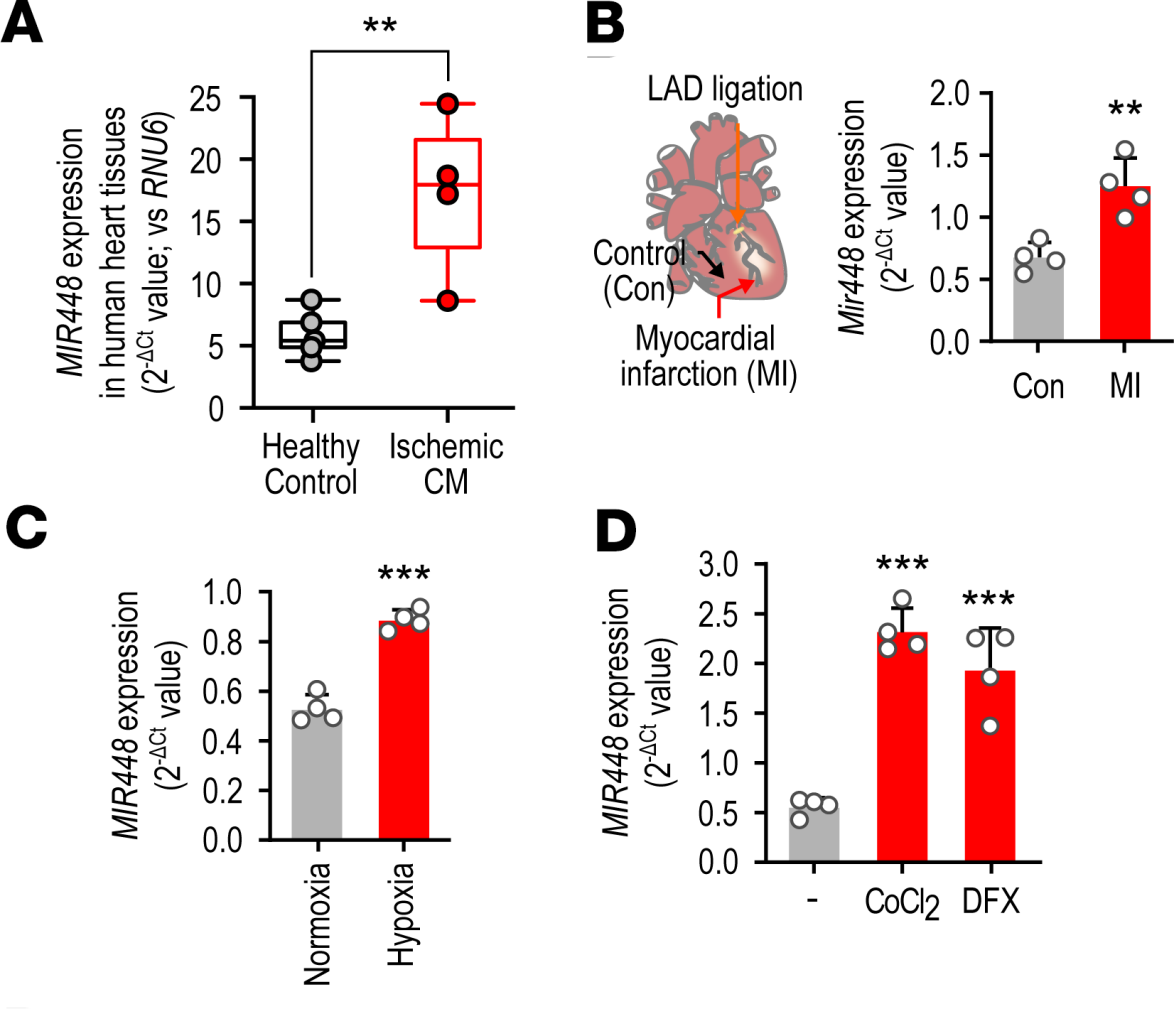
miR-448 increases in ischemia. (**A**) miR-448 expression in human ischemic cardiomyopathy. Left ventricle tissue was obtained from heart failure patients with ischemic cardiomyopathy. (healthy control *n* = 5, ischemic cardiomyocyte [CM] *n* = 4). (**B**) miR-448 expression in murine myocardial infarction (MI). Left ventricle tissue was obtained from the peri-infarct and distal regions for comparison. Data are represented as the mean ± SD or mean + SD of 4–5 samples. **P < 0.01 (when compared between indicated groups by Student’s t test). (**C**) Effect of hypoxia on the miR-448 level in CMs. RL14 cells were incubated in normoxic (21% O_2_) and hypoxic (2% O_2_) conditions for 6 hours. (**D**) Effect of hypoxia-mimetic media on the miR-448 level in CMs. RL14 cells were stimulated with cobalt chloride (CoCl_2_) and desferrioxamine (DFX) for 24 hours. Data are represented as the mean + SD of 4 independent experiments. ***P < 0.001 (when compared between indicated groups by Student’s *t* test).

Cardiac miR-448 expression was significantly increased in heart tissues from patients with ischemic cardiomyopathy compared with healthy controls. miR-448 level scaled with arrhythmic risk (Supplemental Figure 1A; supplemental material available online with this article; https://doi.org/10.1172/jci.insight.140759DS1). A similar upregulation was noted in mouse heart tissue taken from the myocardial infarction (MI) border region when compared with the control remote region (Figure 1, A and B). RL14 human cardiomyocytes (CMs) were used to confirm hypoxia could increase miR-448. Compared with normoxia (21% O_2_), incubating with hypoxia (2% O_2_) for 6 hours caused an increase in the levels of both the mature and precursor form of miR-448 (Figure 1C and Supplemental Figure 1B). miR-448 normoxic levels were comparable to those obtained in vivo under control conditions, and similar changes in miR-448 in response to changes in O_2_ tension were noted in acutely isolated adult CMs (Supplemental Figure 1C). miR-448 expression was also increased by the hypoxia-mimetic chemicals, cobalt chloride (CoCl_2_), desferrioxamine (DFX), and dimethyloxalylglycine (DMOG) (Figure 1D and Supplemental Figure 1D).

### SCN5A was a direct target of miR-448.

**Figure 2:**
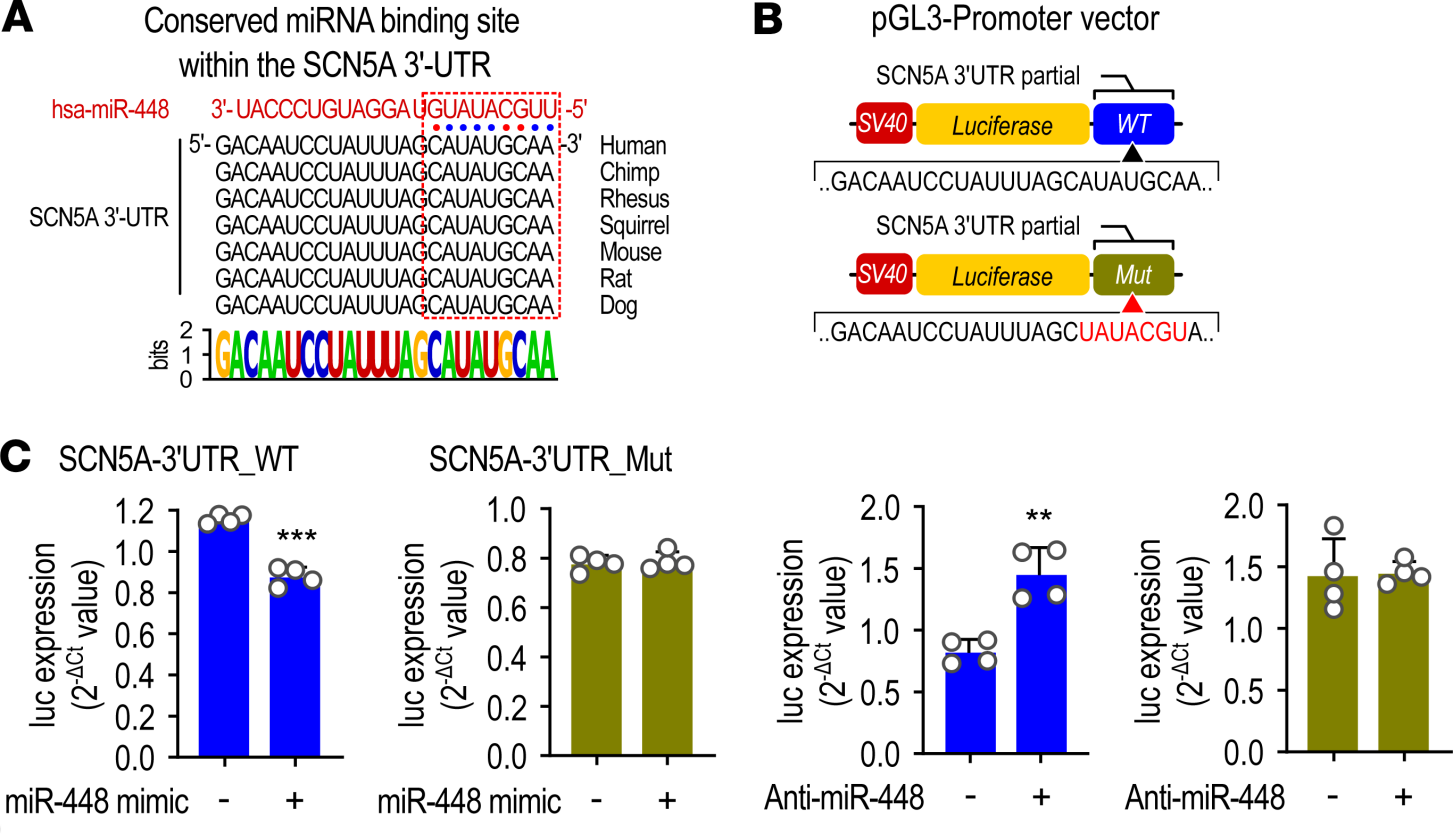
*SCN5A* is a direct target of miR-448. (**A**) Conserved miR-448 binding site within *SCN5A* 3′-UTR. (**B**) Diagram of luciferase reporter constructs. The WT or mutant (Mut) *SCN5A* 3′-UTRs were inserted downstream of the luciferase gene of the pGL3-promoter vector. (**C**) Effect of miR-448 on luciferase mRNA expression in human embryonic kidney 293T (HEK293T). Cells were transfected with WT or mutation plasmid DNA, and then miR-448 mimic or inhibitor were transfected into the cells (10 nM). Data are shown as the mean + SD of 4 independent experiments. **P < 0.01, ***P < 0.001 (when compared between indicated groups by Student’s *t* test).

Sequence analysis revealed a complementary binding sequence for miR-448 within the 3′-UTR of *SCN5A* mRNA. This binding site is highly conserved in many mammals, including human and mouse (Figure 2A). WT or DNA constructs with a mutation of the binding site for miR-448 in 3′-UTR of *SCN5A* were used to confirm *SCN5A* as a target of miR-448. The DNA construct carried the SV40 promoter followed by a luciferase gene. Following luciferase was a partial *SCN5A* 3′-UTR that contained WT or mutant sequences for the miR-448 consensus binding site (Figure 2B). Therefore, if miR-448 bound to and regulated the *SCN5A* 3′-UTR site, luciferase expression would also be regulated concomitantly. EGFP expressing DNA was transfected together with the *SCN5A* 3′-UTR construct, and EGFP was used as an expression control. As expected, luciferase mRNA levels were decreased by the miR-448 mimic and increased by the miR-448 inhibitor. Moreover, these effects did not appear in cells transfected with a DNA construct containing a mutation at the miR-448 binding site (Figure 2C). These results demonstrate that *SCN5A* is a direct target of miR-448.

### SCN5A expression was regulated by miR-448.

**Figure 3:**
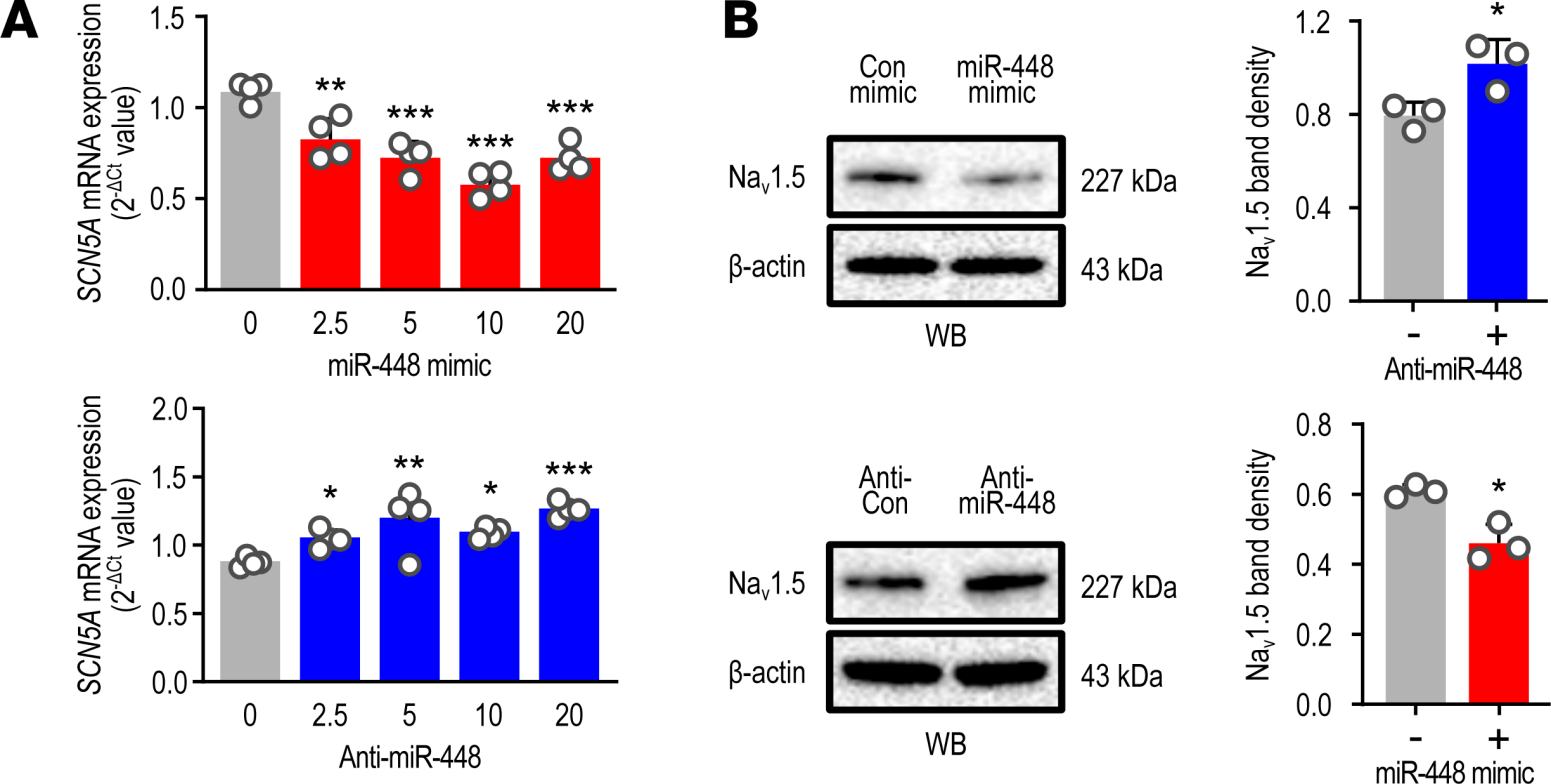
*SCN5A* is regulated by miR-448. (**A**) Effect of miR-448 on the *SCN5A* mRNA level in CMs. Cells were transfected with mimic or anti miR-448 and then incubated for 24 hours (2.5, 5, 10, and 20 nM). Data are represented as the mean + SD of 3–4 independent experiments. One-way ANOVA with Sidak’s multiple-comparison test was performed to determine the *P* value. * *P* < 0.05, **P < 0.01, ***P < 0.001 (represent comparison of mimic or anti-miR-448 to the control group). (**B**) Effect of miR-448 on the protein level of *SCN5A* in CMs. Cells were transfected with mimic or anti-miR-448 (10 nM) and then incubated for 24 hours. Representative Western blots (left) and bar graph (right) representing the quantitative Western blot analysis of Na_v_1.5. Data are represented as the mean + SD of 3–4 independent experiments. *P < 0.05 (when compared between indicated groups by Student’s *t* test).

Next, we investigated whether miR-448 regulates the expression and function of Na_v_1.5 (encoded by *SCN5A*). In RL14 human CMs, *SCN5A* mRNA expression was reduced by the miR-448 mimic and induced by anti-miR-448 (inhibitor) (Figure 3A). Na_v_1.5 protein level was also regulated by treatment of miR-448 mimic or an inhibitor in a manner consistent with miR-448 regulation of *SCN5A* mRNA (Figure 3B).

**Figure 4:**
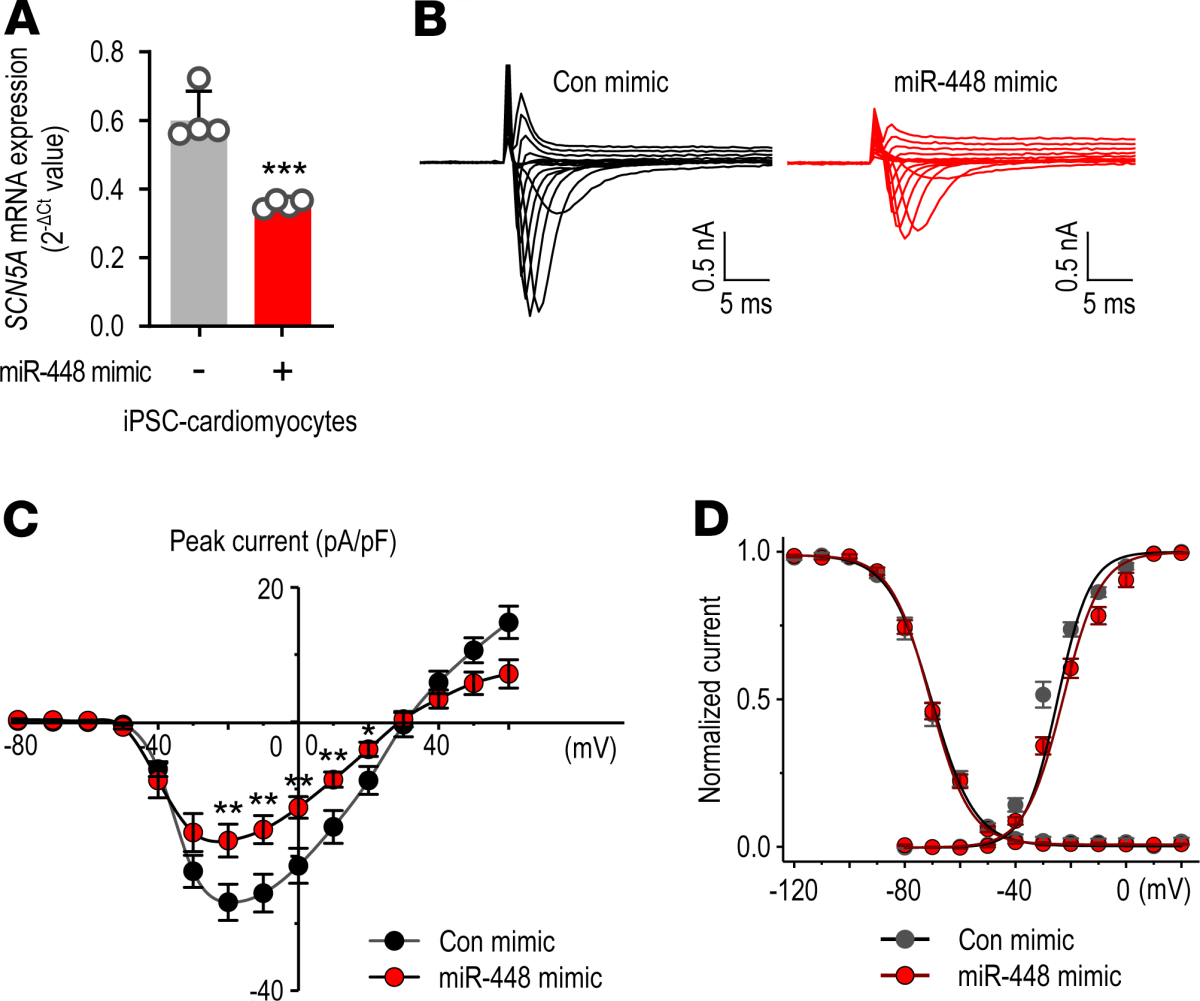
Sodium channel currents are reduced by miR-448 mimic in human iPSC-CMs. (**A**) Effect of miR-448 mimic on the *SCN5A* mRNA level in iPSC- CMs. Cells were transfected with miR-448 mimic (10 nM) and then incubated for 24 hours. (**B**) Representative whole-cell sodium current traces in response to increasing step depolarizations from either control (black) or miR-448 mimic-transfected iPSC-CMs (red). (**C**) Average sodium current–voltage relationship of voltage-dependent sodium channels from either control (black)- or miR-448 mimic (red)-transfected iPSC-CMs. (**D**) Average voltage-dependence of activation and steady-state inactivation in control (black) and miR-448 mimic-transfected iPSC-CMs (red). For the activation curve, normalized peak conductance was plotted as a function of the membrane potential. For the inactivation curve, peak sodium currents were normalized to maximum values in each cell and plotted as a function of the voltage of the conditioning step. Data are represented as the mean + SD or mean ± SEM. *P < 0.05, **P < 0.01, ***P < 0.001 (when compared between indicated groups by Student’s *t* test).

To determine the effect of miR-448 mimic on the sodium current, we used human iPSC-CMs. The *SCN5A* mRNA level was reduced by transfection of the miR-448 mimic (Figure 4A). Similar to *SCN5A* mRNA levels, the peak sodium current in miR-448 mimic transfected iPSC-CMs was reduced compared with control infection (Figure 4, B and C). Channel macroscopic activation or inactivation gating was not altered by control or the miR-448 mimic infections (Figure 4D). Taken together, these results indicated that miR-448 could control the Na_v_1.5 current level in addition to the *SCN5A* mRNA level.

### Hypoxia-induced miR-448 controlled SCN5A.

To investigate the regulation of *SCN5A* in the hypoxic condition, RL14 cells were stimulated with DFX. *SCN5A* mRNA expression was decreased by DFX stimulation in a dose- and time-dependent manner, and Na_v_1.5 protein level also decreased (Supplemental Figure 2).

**Figure 5:**
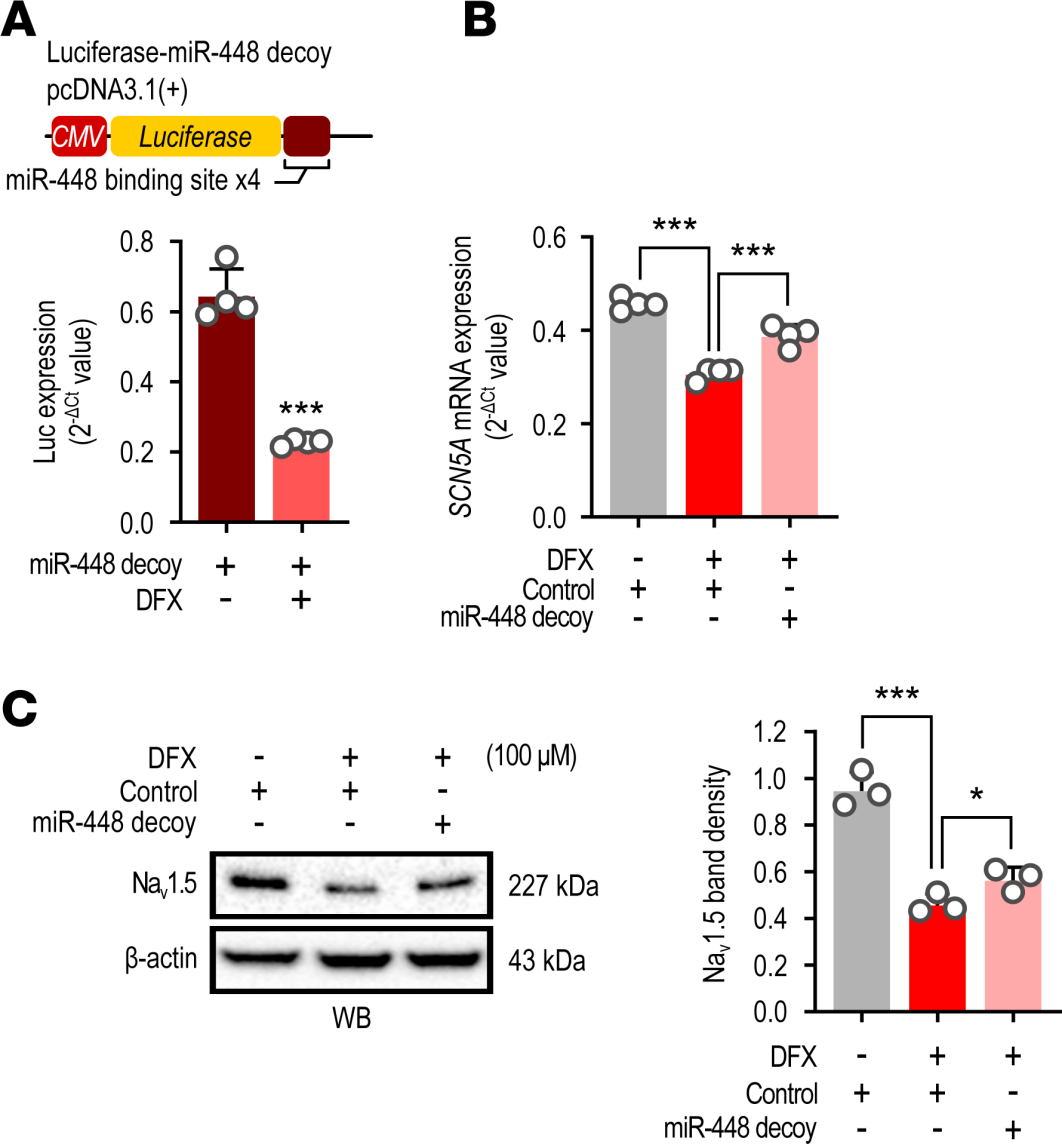
*SCN5A* is regulated by hypoxia-induced miR-448. (**A**) Diagram of luciferase reporter constructs. The miR-448 binding sequences were inserted downstream of the luciferase gene of the pGL3-promoter vector. Cells were transfected with miR-448 decoy and then luciferase (LUC) expression was checked in the presence or absence of DFX. (**B**) Effect of miR-448 decoy on *SCN5A* mRNA expression reduced by simulated hypoxia in CMs. Cells were transfected with miR-448 decoy and then stimulated with DFX for 6 hours. (**C**) Effect of miR-448 decoy on the DFX-induced Na_v_1.5 protein in CMs. Cells were transfected with miR-448 decoy and then stimulated with DFX for 24 hours. Representative Western blots (top) and bar graph (bottom) representing the quantitative Western blot analysis of Na_v_1.5. Data are represented as the mean + SD of 3–4 independent experiments. *P < 0.05, ***P < 0.001 (when compared between indicated groups by Student’s *t* test or 1-way ANOVA with Dunnett’s multiple-comparison test).

DNA construct with 4 miR-448 binding sites following the luciferase gene (miR-448 decoy) was used to inhibit the effect of miR-448 on *SCN5A*. EGFP DNA was used as a transfection control. Reduced luciferase mRNA level in DFX-stimulated RL14 cells confirmed that the miR-448 decoy was effective in acting competitively against miR-448 increased by DFX stimulation (Figure 5A). The reduced mRNA expression of *SCN5A* was rescued by the miR-448 decoy treatment (Figure 5B). We confirmed that the Na_v_1.5 protein level was also rescued in the cells transfected with the miR-448 decoy (Figure 5C). These results indicated that *SCN5A* expression can be regulated by hypoxia-induced miR-448.

### HIF-1α and NF-κB regulated miR-448 in hypoxia.

**Figure 6:**
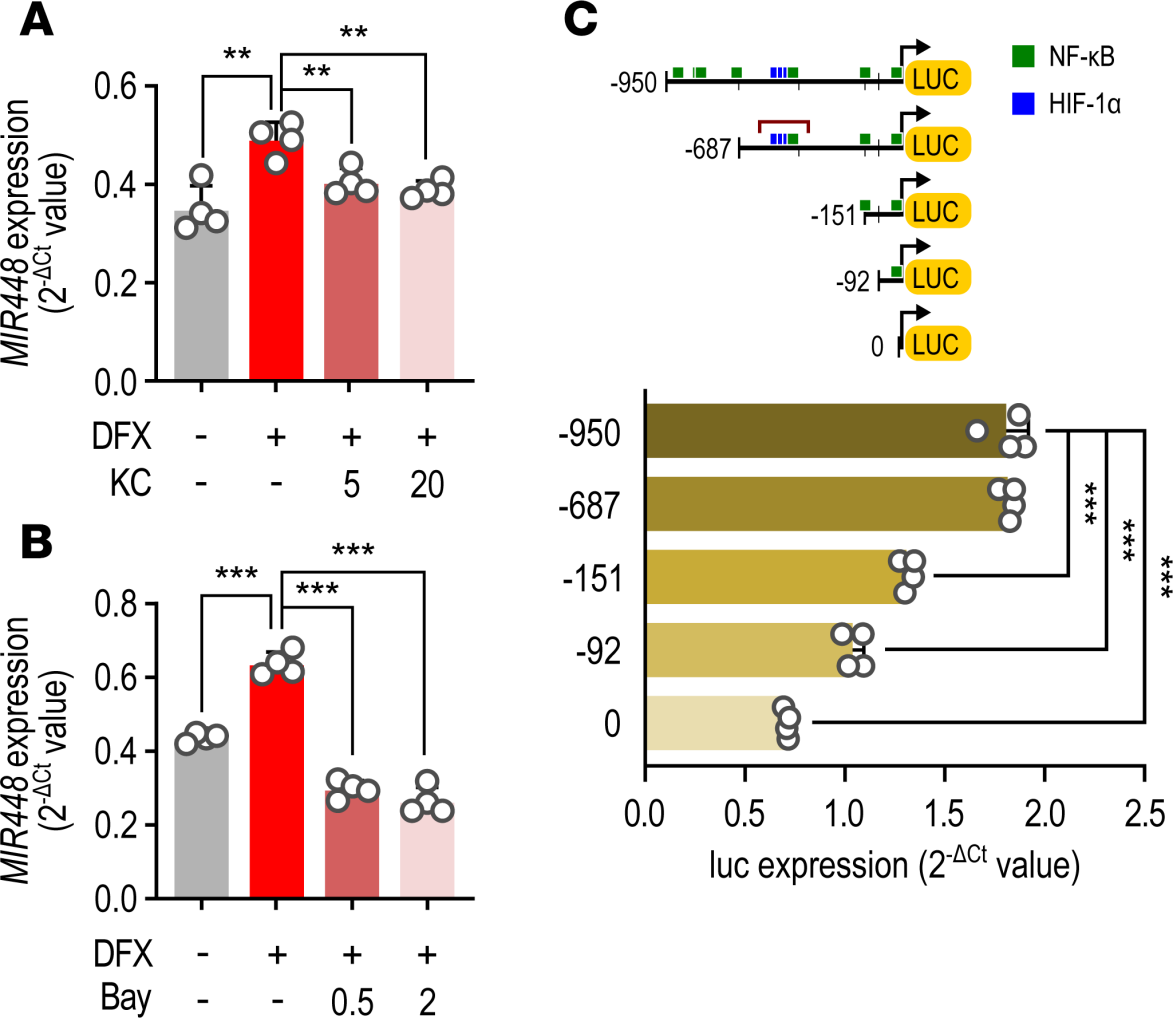
HIF-1α and NF-κB are upregulators of *MIR448* in hypoxia. (**A**) Effect of KC7F2, a selective HIF-1α transcription inhibitor, on the induction of miR-448 by DFX in CMs. (**B**) Effect of Bay11-7082, a NF-κB inhibitor, on the induction of miR-448 by DFX in CMs. Cells were treated with HIF-1α or NF-κB inhibitors in a dose-dependent manner (5, 20 or 0.5, 2 μM, respectively) for 30 minutes and then were stimulated with DFX for 6 hours. (**C**) Predicted binding sites for HIF-1α (blue squares) or NF-κB (green squares) are within 1 kb upstream of the *MIR448* transcriptional initiation site. Diagrams show luciferase reporter constructs with 1 kb promoter *MIR448* region or series of deletion mutants. Data are represented as the mean + SD of 4 independent experiments. **P < 0.01, *** *P <* 0.001 (when compared between indicated groups by Student’s *t* test or 1-way ANOVA with Sidak’s multiple-comparison test).

The *MIR448* gene is located in an intron of the HTR2C gene, but *MIR448* is regulated independently from HTR2C (17). Therefore, we investigated how hypoxia regulated miR-448 expression. A transcription factor binding site prediction tool (18) suggested binding sequences for HIF-1α and NF-κB within 1 kb from the transcription initiation site of *MIR448*. HIF-1α and NF-κB are well-known hypoxia response factors (19–21). DFX treatment of RL14 CMs for 6 hours increased the protein level and the translocation into the nucleus of HIF-1α and NF-κB (Supplemental Figure 3). KC7F2 (KC; HIF-1α inhibitor) or Bay11-7028 (Bay; NF-κB inhibitor) inhibited the increase of miR-448 by DFX (Figure 6, A and B). To confirm this apparent transcriptional regulation, a series of 3 deletion mutants, –687, –151, or –92 bp promoter regions from the *MIR448* transcriptional initiation site, were inserted upstream of the luciferase gene in the pGL3-Basic vector. The binding sites for HIF-1α and NF-κB present in the –950 bp promoter region are indicated by blue and green square boxes, respectively. Deletion of the promoter region from nt –687 to –151 produced a dramatic reduction in transcriptional activity (Figure 6C), indicating that this site, which contains HIF-1α and NF-κB binding elements, is critical for the regulation of *MIR448* expression. These results suggest that hypoxia-induced HIF-1α and NF-κB are important in regulating *MIR448* expression.

### RE1 silencing transcription factor suppressed miR-448.

**Figure 7:**
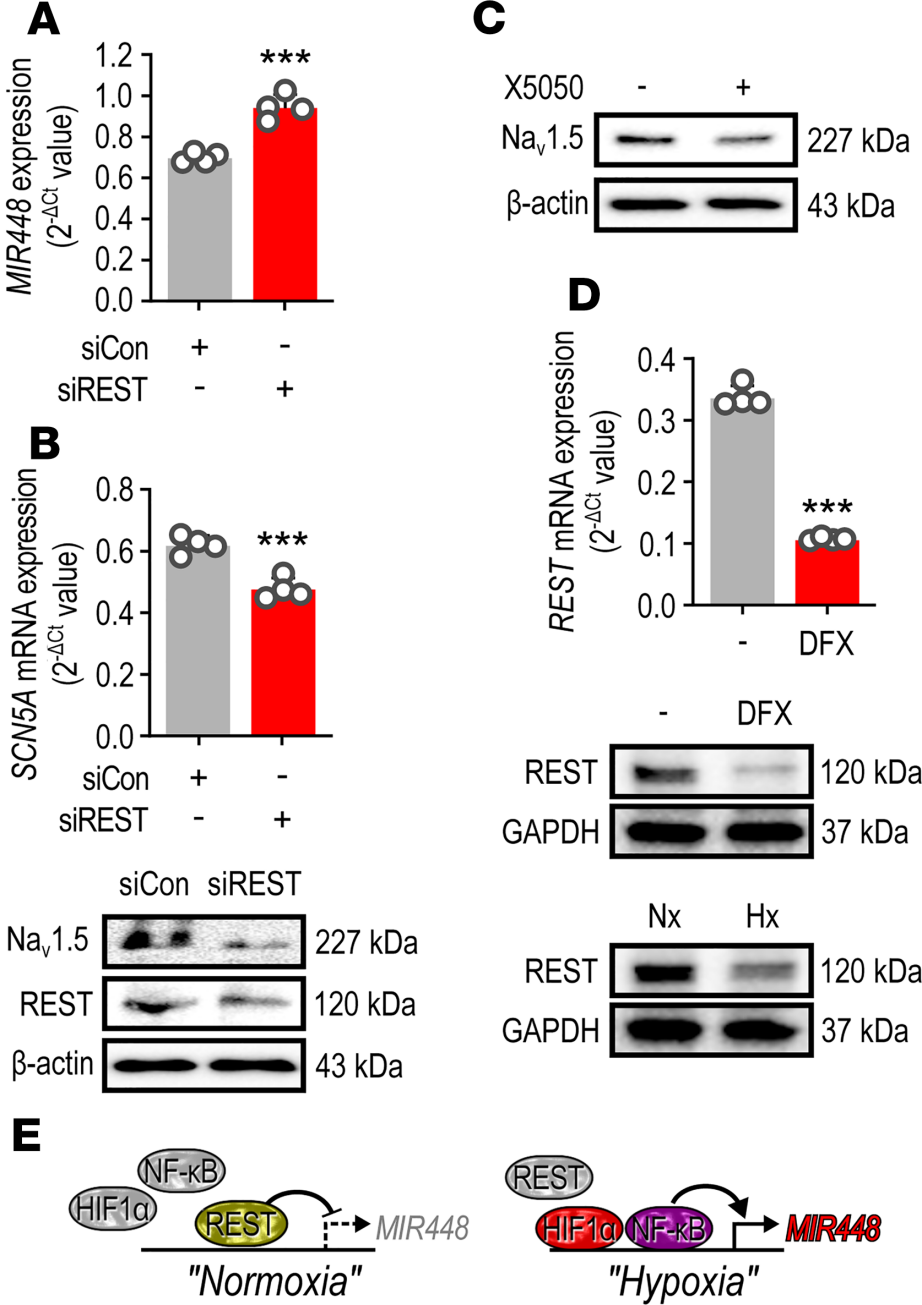
Ischemia relieves RE1 silencing transcription factor repression of *MIR448*. (**A**) Effect of REST gene silencing on the miR-448 level in CMs. Cells were transfected with control or RE1 silencing transcription factor (REST) siRNA for 24 hours. (**B**) Effect of REST gene silencing on the mRNA (top) and protein (bottom) level of *SCN5A* in CMs. Cells were transfected with control or REST siRNA for 24 hours. (**C**) Effect of X5050, a REST inhibitor, on the protein level of Na_v_1.5 in CMs. Cells were treated with X5050 for 24 hours. (**D**) Effect of hypoxic condition on the mRNA (top) and protein (bottom) level of REST. Cells were stimulated with DFX for 6 hours or were incubated with normoxia or hypoxia for 6 hours. (**E**) Diagram showing *MIR448* transcriptional regulation by HIF-1α, NF-κB, and REST in normoxia and hypoxia. Data are represented as the mean + SD of 4 independent experiments. ***P < 0.001 (when compared between indicated groups by Student’s *t* test).

Within the 1 kb *MIR448* promoter was a predicted RE1 silencing transcription factor (REST) binding site. REST was initially identified as a transcriptional repressor that regulates neuronal genes in nonneuronal tissues (22). Interestingly, REST regulates other genes in response to changes in O_2_ tension by direct binding to an RE1 (also known as NRSE) site on their promoter regions (23, 24). In normoxia, *MIR448* expression was increased by REST gene silencing (Figure 7A), and *SCN5A* mRNA and Na_v_1.5 protein levels decreased. Moreover, Na_v_1.5 protein level was decreased by the REST specific inhibitor, X5050 (Figure 7, B and C). REST mRNA and protein were decreased by hypoxia (Figure 7D). These results indicated that miR-448 expression is inhibited by REST in normoxia and that REST, together with HIF-1α and NF-κB, is an important transcription factor regulating miR-448 in ischemia.

### miR-448 inhibition raised Na_v_1.5 and reduced arrhythmia in MI.

Ischemia is known to reduce Na_v_1.5, and reduced Na_v_1.5 is arrhythmogenic. Ischemia-induced miR-448 contributes to the reduction in Na_v_1.5. Therefore, we tested whether inhibition of miR-448 could raise Na_v_1.5 and reduce arrhythmic risk in ischemic cardiomyopathy. Mice underwent left anterior coronary artery (LAD) ligation to create myocardial ischemia and infarction. A miR-448 sponge was used to inhibit miR-448 function. In human CMs, sponge treatment rescued *SCN5A* mRNA levels decreased by miR-448 treatment, indicating the efficacy of this sponge approach (Supplemental Figure 4).

**Figure 8:**
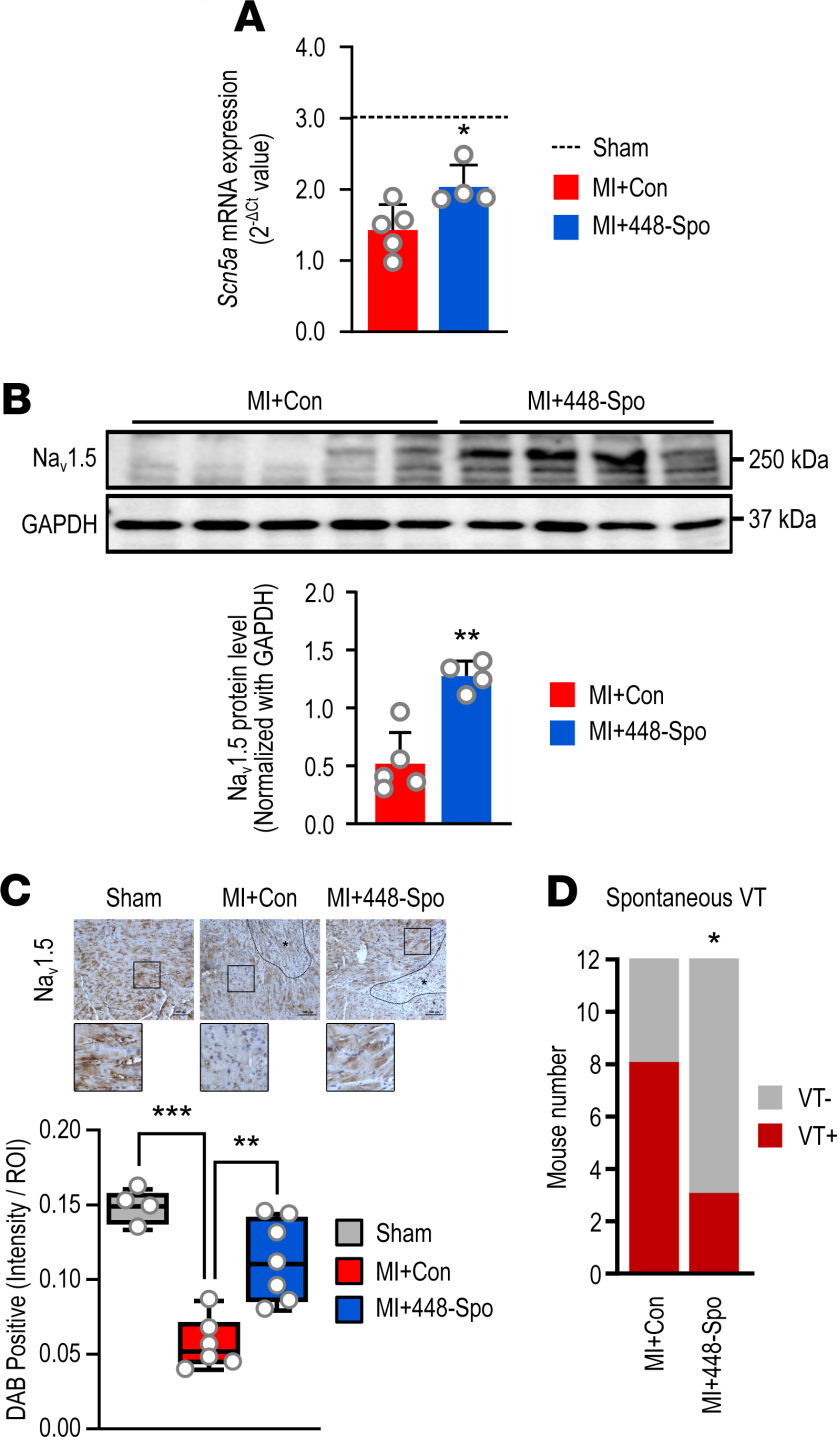
Blocking of miR-448 improves Na_v_1.5 levels and arrhythmic risk after MI. (**A**) Effect of miR-448 antagonism on cardiac *SCN5A* mRNA level after MI. The heart tissues were collected from MI+Con or MI+448-Spo. (**B** and **C**) Effect of miR-448 antagonism on protein level of cardiac Na_v_1.5 after MI. The heart tissues were collected from MI+Con or MI+448-Spo. IHC localization of Na_v_1.5 antigen done using formalin-fixed, paraffin-embedded heart tissues. Tissue sections were incubated with Na_v_1.5 antibody. Positively stained cells were evaluated using Image J analysis. (**D**) The number of mice in each group with or without ventricular tachycardia (VT). Data are represented as the mean + SD or mean ± SD. *P < 0.05, **P < 0.01, ***P < 0.001 (when compared between indicated groups by Student’s *t* test or 1-way ANOVA with Sidak’s multiple-comparison test).

To test the role of miR-448 in regulating *SCN5A* expression in vivo, we injected MI mice with either AAV9-Control (Con) or AAV9-miR-448 sponge (448-Spo) viral particles 2 weeks before coronary artery ligation. To confirm gene expression in the heart through AAV9 injection, GFP expression was examined in the heart tissue of mice treated with an AAV9 vector encoding the GFP gene. The green fluorescence ratio increased compared with WT with AAV9-GFP treatment, and the GFP level also increased in the heart treated with AAV-GFP (Supplemental Figure 5). After 2 weeks of LAD ligation, mouse hearts were harvested to evaluate *SCN5A* expression by real-time quantitative PCR (RT-qPCR), Western blot, and IHC. Mice echocardiography was performed to confirm the MI) by LAD ligation. The ejection fraction (EF) of the MI + AAV9-Con group was reduced compared with the sham group, indicating that MI was present. AAV9-448-Spo treatment did not affect EF% change (Supplemental Figure 6). We estimated infarct size by echocardiography. Infarct size was unchanged between mice with AAV9-Con and AAV9-448-Spo. The cardiac mRNA level of *SCN5A* was increased in mice injected with 448-Spo compared with those injected with the Con (Figure 8A). Moreover, the Na_v_1.5 protein level was increased in mice injected with 448-Spo (Figure 8, B and C). Among the 24 mice with ventricular events, ventricular tachycardia (VT) was detected in 8 out of 12 mice in the Con group, whereas VT was detected only in 3 out of 12 mice in the 448-Spo group (*P* < 0.05) (Figure 8D). These results indicated that miR-448 inhibition after MI raises Na_v_1.5 and lowers the risk of severe ventricular arrhythmia. We did not study miR-448 antagomir regional effects on the heart; however, global application of the antagomir had an overall antiarrhythmic effect.

## Discussion

Arrhythmia is among the leading causes of death from ischemic cardiomyopathy. Arrhythmias are thought to be caused by ventricle structural and electrical remodeling, consisting mostly of downregulation of ion channels by a variety of mechanisms (12, 25, 26). These mechanisms include transcriptional, RNA processing, RNA stability, translation efficiency, and posttranslational dysregulation. Reversing the ion channel downregulation can improve arrhythmic risk (5, 9, 12–14, 27) and may represent an alternative approach to ion channel blocking antiarrhythmic drugs.

*SCN5A* alterations, either up- or downregulation, are known to cause arrhythmias, and *SCN5A* is downregulated in ischemic cardiomyopathy (27–29). Nevertheless, the mechanisms whereby ischemia causes a reduction in *SCN5A* have not been fully explored but include mRNA destabilization (9, 27). We investigated the mechanism of *SCN5A* mRNA instability during cardiac hypoxia and showed that the level of miR-448 level increased during ischemia, miR-448 bound to *SCN5A* mRNA, and this binding reduced *SCN5A* mRNA, protein, and current. Moreover, we showed that HIF-1α and NF-κB upregulation and REST downregulation in response to hypoxia controlled miR-448 levels. As expected, downregulation of *SCN5A* in ischemic cardiomyopathy was associated with increased arrhythmic risk, and miR-448 inhibition could restore Na_v_1.5 levels and reduce arrhythmic risk. Although interpretation of mouse telemetry for ventricular events can be subject to some error, the correlation of miR-448 with arrhythmic risk in humans suggests that any error did not affect the qualitative result. In this study, even though cardiac sodium channel mRNA, channel protein, and sodium current changes appeared inversely related to arrhythmic risk, we cannot rule out some differences between channel mRNA, protein, and current in response to miR-448.

Other miRs are known to regulate *SCN5A*. Previous studies have demonstrated that *SCN5A* can be controlled by miRs targeting the gene coding region and the 3′-UTR. Recent studies have shown that miR-24 and miR-1270 can regulate expression by directly binding to binding sites created by single nt polymorphisms of *SCN5A* (29, 30). Previous reports have shown that miR-192-5p increases in atrial fibrillation and inhibits *SCN5A* expression (31). Furthermore, it has been previously reported that miR-200c directly or indirectly regulates various cardiac genes, including *SCN5A* (32). Here, we showed that miR-448 directly bound to the *SCN5A* 3′-UTR and regulated the Na_v_1.5 current.

We have shown previously that loss of Hu antigen R (HuR; encoded by *ELAVL1*) can destabilize *SCN5A* and contribute to arrhythmic risk in cardiomyopathy (9, 27). The binding site for miR-448 is located adjacent to the HuR binding site in the 3′-UTR of *SCN5A*. HuR is a well-known mRNA stabilization factor binding AU-rich domains in the target mRNA, and some of the effects of HuR could be explained by its interplay with miR-448. Binding of HuR may prevent the miR-448 interaction with the 3′-UTR.

Our results show that miR-448 increases in hypoxic conditions, and the miR-448 promoter region analysis shows that there are binding sites for hypoxia-related factors. The response to hypoxic stress is coupled tightly to the interaction between HIF-1α and NF-κB signaling. Maximal HIF-1α expression depends on transcriptional regulation by NF-κB and posttranslational regulation by hypoxia (33–35). Our results showed that HIF-1α and NF-κB binding sites were –530 to –450 upstream from the miR-448 gene transcription initiation site and were important in regulating miR-448 expression during hypoxia.

REST can regulate genes in response to changes in O_2_ tension. Previous reports have shown that the reduction of REST contributes to gene upregulation in hypoxia (23, 24, 36, 37). Our *MIR448* promoter analysis showed that the binding sequence for REST is within the 1 kb region of the *MIR448* promoter, and that REST gene silencing in normoxia leads to an increase in miR-448 expression and a decrease in *SCN5A* expression. Therefore, there appears to be an interplay of relief of REST inhibition and increased HIF-1α and NF-κB induction to regulate miR-448 and, therefore, *SCN5A*. Consensus binding sequence analysis has shown that REST may also be a target of miR-448, suggesting a more complex negative feedback loop.

**Figure 9:**
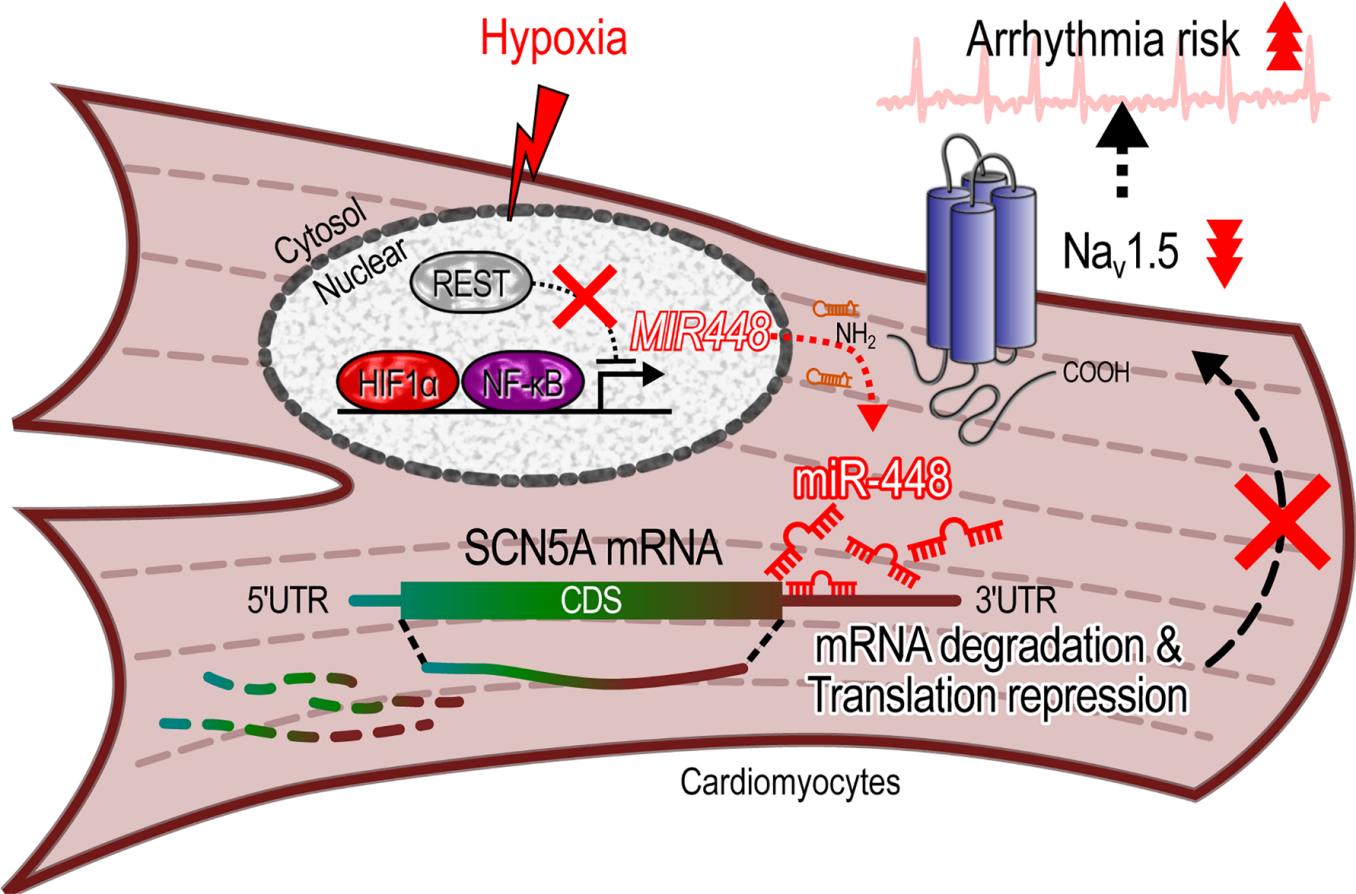
Summary of the effect of hypoxia on miR-448, *SCN5A*, and arrhythmic risk.

In summary, miR-448 negatively regulated the cardiac sodium channel by direct binding to 3′-UTR of *SCN5A* mRNA. The effect of miR-448 increased in ischemia. Inhibition of miR-448 raised Na_v_1.5 and reduced arrhythmic risk after MI, suggesting a new paradigm in antiarrhythmic therapy (Figure 9).

## Methods

### Cell culture and transfection.

Human fetal CM cell line RL14 cells (ATCC RL-14, ATCC) were grown in DMEM/F-12 nutrient mixture (GE Healthcare Life Sciences) supplemented with 12.5% (v/v) fetal bovine serum (Gibco) and penicillin-streptomycin (10,000 U/mL; Gibco). Human embryonic kidney 293T (HEK293T) cells (ATCC CRL-3216, ATCC) were maintained in DMEM high glucose supplemented with 10% fetal bovine serum and penicillin-streptomycin. Human-induced pluripotent stem cell–CMs (hiPSC-CMs) were obtained from Cellular Dynamics International. hiPSC-CMs were seeded and maintained using iCell CM Plating Medium and Maintenance Medium (Cellular Dynamics International). For hypoxic conditions, the cells were cultured in an hypoxic incubation chamber (STEMCELL Technologies) using preincubated culture media or were treated with hypoxic-mimetic chemicals, CoCl_2_, DFX, and DMOG (MilliporeSigma) (38, 39).

### Cell transfection.

Syn-hsa-miR-448 miScript miRNA mimic, anti-hsa-miR-448 miScript miRNA inhibitor, and its negative controls were purchased from Qiagen. REST siRNA and its negative control were obtained from Integrated DNA Technologies (IDT). The cells were transfected using HiPerFect Transfection Reagent (Qiagen) following the recommendations of the manufacturer. Plasmid DNA was transfected into cultured cells using SuperFect transfection reagent (Qiagen) following the manufacturer’s protocol.

### Plasmid constructions.

Gene fragments for *SCN5A* 3′-UTR with or without mutations at the miR-448 binding site were obtained from IDT. They were cloned into pGL3-Promoter vectors downstream of the luciferase ORF to create pGL3-Promoter-Luciferase *SCN5A* 3′-UTR WT or mutation (Mut) vectors. A gene fragment for miR-448 acting as a decoy was obtained from IDT and was cloned into pcDNA3.1 (+) luciferase vector downstream of the luciferase ORF to create a pcDNA3.1(+)-luciferase-miR-448 decoy vector. The miR-448 decoy sequence was designed and confirmed using the “miRNAsong” web tool (40). A gene fragment for a 1 kb promoter region of miR-448 was obtained from IDT and cloned into the pGL3-Basic vector upstream of the luciferase ORF to create a pGL3-*MIR448* promoter-luciferase vector. Then, –687, –151, and –92 bp DNA fragments were amplified by PCR with specific primers, and the fragments were cloned into the pGL3-Basic vector. All DNA constructs were confirmed by DNA sequencing. Each DNA construct was transfected into CMs, and the cells were stimulated with DFX for 6 hours. Luciferase mRNA level was detected by qPCR. The mRNA level of luciferase was normalized with the mRNA level of EGFP, which was cotransfected as a control.

### RNA preparation and RT reverse transcription PCR.

Total RNA was prepared using RNeasy Plus Mini Kit or miRNeasy Mini Kit (Qiagen) according to the manufacturer’s instructions. Reverse transcription was performed with a First-Strand cDNA Synthesis Kit (Promega). cDNA for miRNA detection was generated by the miScript II RT Kit (Qiagen). RT reverse transcription quantitative PCR was performed with SYBR Green PCR Master Mix (Thermo Fisher Scientific) using the 7500Fast Real-Time PCR system (Thermo Fisher Scientific) and miScript SYBR Green PCR Kit with miScript Primer Assays to detect miRNA expression. The primer sequences were as follows: *SCN5A* (F 5′-TGGTTGTCATCCTCTCCATCGT-3′, R 5′-ATGAGGGCAAAGAGCAGCGT-3′), and *GAPDH* (F 5′-GAAGGTGAAGGTCGGAGTCAAC-3′, R 5′-CAGAGTTAAAAGCAGCCCTGGT-3′). Hs_miR-448_1 miScript Primer Assay (5′UUGCAUAUGUAGGAUGUCCCAU; Qiagen) was used for detecting miR-448, and the Hs_RNU6-2_11 miScript Primer Assay (Qiagen) was used as an endogenous control. The relative fold change was calculated by the 2^–ΔΔCt^ method, and the measurements were normalized with respect to the endogenous control (*RNU6, GAPDH*).

### Western blot.

Cells were washed twice with ice-cold PBS and disrupted in cell lysis buffer (Cell Signaling Technology) with Protease and Phosphatase Inhibitor Cocktail (Thermo Fisher Scientific) on ice for 30 minutes. Cell lysates were centrifuged at 20,854*g* for 15 minutes at 4°C, and the resultant supernatants were subjected to Western blotting (see complete unedited blots in the supplemental material). The total protein concentration was quantified using the Pierce BCA Protein Assay Kit (Thermo Fisher Scientific). Next, proteins were separated by electrophoresis on 4%–15% Mini-PROTEAN TGX Precast Protein Gels (Bio-Rad), after which samples were transferred onto a PVDF membrane. The membrane was treated with 5% skim milk for 1 hour and incubated with Na_v_1.5 antibody (1:1000 dilution; ab56240, Abcam), HIF-1α (1:1000 dilution; ab72775, Abcam), NF-κB (1:1000 dilution; ab32536, Abcam), REST (1:1000 dilution; ab21635, Abcam), GAPDH (1:1000 dilution; ab9484, Abcam), and β-actin (1:5000 dilution; A5441, MilliporeSigma) overnight at 4°C. After TBST washing, the membrane was incubated with HRP-conjugated secondary antibody (1:5000) for 90 minutes at room temperature. The proteins were visualized with Pierce ECL Western Blotting Substrate (Thermo Fisher Scientific) using the ChemiDoc XRS+ System (Bio-Rad). The images were analyzed using ImageJ software to measure band density, then band density was normalized with β-actin from 3 independent experiments.

### Electrophysiology.

hiPSC-CMs were trypsinized (0.25% trypsin-EDTA; Invitrogen, Thermo Fisher Scientific) for 10 minutes and plated in 35 mm culture dishes at a cell density of approximately 100 cells per dish on the day before the experiments. Na^+^ channel currents were measured by using the whole-cell patch-clamp technique in the voltage-clamp configuration at room temperature. hiPSC-CMs were not selected by action potential morphology, but the differentiation technique resulted in predominantly ventricular-like cells. To measure Na^+^ channel currents, pipettes (2–4 MΩ) were filled with a pipette solution containing (in mmol/L): CsCl 80, cesium aspartate 80, EGTA 11, MgCl_2_ 1, CaCl_2_ 1, HEPES 10, and Na2ATP 5 (adjusted to pH 7.4 with CsOH). The bath solution consisted of (in mmol/L): NaCl 10, NMDG 100, TEA-Cl 20, CsCl 5, CaCl_2_ 2, MgCl_2_ 1.2, HEPES 10, and glucose 5 (adjusted to pH 7.4 with CsOH). The holding potential was -100 mV. A voltage step protocol, ranging from –80 to +60 mV with steps of 10 mV, was applied to establish the presence of the Na^+^ channel currents. The peak current density was used to plot current-voltage (I-V) curves. Nifedipine (10 μM, MilliporeSigma) was added in the bath solution to block L-type Ca^2+^ channel currents. Steady-state activation and inactivation were characterized by Boltzmann functions.

### Human samples.

Deidentified control heart tissue was a gift of JA Wasserstrom of Northwestern University. All control heart tissue was derived from patients who suffered brain death because of a cerebral vascular accident and had no concomitant cardiac conditions before the heart was harvested. Deidentified HF heart tissues were obtained from the Lillehei Heart Institute Tissue Bank at the University of Minnesota. HF patients had a history of ischemic cardiomyopathy that was confirmed by histological specimens prepared concomitantly with acquisition of the specimen. In the control and HF groups, specimens were from the left ventricle. Arrhythmic status was determined by record review.

### Mouse cardiac ischemia model.

To create ischemia, mice underwent permanent LAD ligation as we previously performed (27). Eight-week-old C57BL/6 mice of both genders were randomly divided into the MI+ adeno-associated virus serotype 9 (AAV9) control group (MI+Con) and MI+AAV9-miR-448 sponge groups (MI+448-Spo). To investigate whether miR-448 inhibition was sufficient to prevent arrhythmia in vivo, AAV9 viral particles (VectorBuilder Inc.) bearing either an empty vector or anti-miR-448 were systemically injected into mice via the right jugular vein at a dose of 5 X 10^11^ viral genomes per animal before LAD ligation.

### Telemetry monitoring.

Twelve randomly selected MI+Con or MI+448-Spo mice were implanted with ETA-F10 transmitters (Data Sciences International). Mice were anesthetized with 4% and maintained on 2% inhaled isoflurane. A skin incision was made in the right abdominal region, and a transmitter was inserted subcutaneously. The 2 electrocardiographic leads were tunneled and positioned under the skin to generate a lead II electrocardiographic configuration. One week after transmitter implantation, electrocardiographic signals were recorded for 24 hours. Heart rate calculations and cardiac rhythm analysis were performed by using Dataquest ART, version 4.1 (Data Sciences International) and LabChart 7 Pro, version 7.3.7 (AD Instruments) (41, 42). A total of 10-hour ECG signals sampled every 3 hours throughout the day were used for offline analysis of ventricular arrhythmic events, including premature ventricular contraction (PVC) and ventricular tachycardia (defined as at least 3 PVCs in succession). ECG signal analysis was performed blinded to treatment.

### IHC.

The heart tissues were fixed in 4% paraformaldehyde (pH 7.4) for 24 hours, embedded in paraffin, and serially sectioned to 5 μm thickness. For the dewaxing process, the paraffin sections were placed at 60°C overnight and then transferred to xylene (10 minutes, 3 times), anhydrous ethanol (3 minutes, twice), 95% ethanol (1 minute, once), 70% ethanol (1 minute, once), and distilled water (2 minutes, once). The tissue sections were heated with citrate buffer (pH 6.0) for 5 minutes at 100°C. After rinsing using washing buffer, the sections were incubated in 3% hydrogen peroxide to quench endogenous peroxidase activity and blocked with 3% BSA (Bio-Rad) to prevent nonspecific antibody binding. Next, the tissue sections were incubated with primary antibodies against GFP (1:200 dilution; sc-9996, Santa Cruz Biotechnology) and Na_v_1.5 (1:200 dilution, ab56240, Abcam) at 4°C overnight and HRP-conjugated secondary antibodies (Bio-Rad) for 30 minutes at room temperature. Finally, the sections were incubated with DAB peroxidase substrate kit (Vector Laboratories) and counterstained with hematoxylin (Vector Laboratories). After permanent mounting, the sections were imaged using a light microscope (Axioscope 7, Zeiss). In captured image, the regions of interest were demarcated using ZEN Pro software (Zeiss). Quantitative analysis was performed using Image Pro Plus software (version 6.0; Media Cybernetics).

### Statistics.

Statistical significance between groups was performed using 2-tailed Student’s *t* tests (paired and unpaired), 1-way ANOVA with Dunnett’s multiple-comparison test, and χ^2^ test where appropriate. For all analyses, a *P* value of less than 0.05 was considered significant. GraphPad Prism software, version 8.0, and R programming software were used to perform all data analyses. All data represent the mean + SD or the mean ± SD.

### Study approval.

All animal protocols used in this study were approved by the IACUC of University of Minnesota. Animal care and interventions were undertaken in accordance with the Guide for the Care and Use of Laboratory Animals (National Academies Press, 2011). Tissue samples and deidentified patient information were obtained by the Biological Materials Procurement Network (BioNet), an Institutional Review Board–approved, centralized human specimen procurement program at the University of Minnesota.

## Author contributions

GJK and SCD conceived and planned the experiments. GJK, AX, and HL carried out the experiments. GJK took the lead in writing the manuscript. SCD supervised the work. All authors provided critical feedback and helped shape the research, analysis, and manuscript.

## Supplementary Material

Supplemental data

## Version 1 10/27/2020

In-Press Preview

## Version 2 12/03/2020

Electronic publication
