# Supplementary material for: MIR448 antagomir reduces arrhythmic risk after myocardial infarction by upregulating the cardiac sodium channel
Source: JCI Insight. 2020 Dec 3;5(23):e140759. doi: 10.1172/jci.insight.140759 (PMC7714400; doi:10.1172/jci.insight.140759)
Supplement: Supplemental data [file jciinsight-5-140759-s099.pdf]

Supplementary Materials

Materials and Methods

Immunofluorescence staining. Cells were seeded onto coverslips and stimulated with DFX for 6 h. Cells were fixed with 4% paraformaldehyde (PFA) for 10 min at room temperature and permeabilized with 0.5% Triton X-100 for 15 min followed by several washes with PBS. After blocking with 3% BSA in PBS at room temperature for 1 h, coverslips were incubated with anti-HIF1α, anti-NF-κB antibodies overnight at 4°C. Excess antibody was removed with PBS and then species-specific secondary antibodies conjugated to goat anti-mouse IgG antibody (Alexa Fluor 488, 1:500 dilution) and donkey anti-rabbit IgG antibody (Alexa Fluor 594, 1:500 Molecular probes, ThermoFisher Scientific) were reacted with the coverslips for 1 h at room temperature. After four washes in PBS, the slides were mounted with mounting solution and visualized at 40X magnification using a Nikon A1R FLIM confocal microscope.

Isolation of mouse adult ventricular cardiomyocyte. Mouse adult ventricular cardiomyocytes (AVCM) were isolated from eight week old C57BL/6 mice. Briefly, heart was excised from anesthetized mice, perfused with perfusion buffer (in mmol/L: NaCl 113, KCl 4.7, Na_2HPO_4 0.6, KH_2PO_4 0.6, $MgSO_4$ 1.2, Phenol Red 0.032, $NaHCO_3$ 12, $KHCO_3$ 10, HEPES 10, taurine 30, and 2-3-butanedione monoxime 10) and digested with collagenase II (Worthington Biochemical Co. Lakewood, NJ). AVCM were washed with control buffers (in mmol/L: NaCl 133.5, KCl 4, Na_2HPO_4 1.2, HEPES 10, and $MgSO_4$ 1.2) with serially increasing Ca^{2+} concentrations (0.2, 0.5, and 1 mmol/L). AVCM were incubated in modified Eagle's medium (MEM medium) with 1% insulin-transferrin-selenium, 0.1% bovine serum albumin, 1% glutamine, and 1% penicillin/streptomycin in a 95% O_2/5% CO_2 incubator at 37ºC for 1 h prior to experiments.

Echocardiography Imaging and Analysis. A Vevo 2100 echocardiographic imaging system (Fujifilm VisualSonics Inc.) with MS550 probe (30 μm resolution) was used to analyze cardiac function in mice. Isoflurane (1–2%) was used to maintain the mice in anesthetic condition for image acquisition. After removing the hair on the chest using Nair (hair removal cream), all images were obtained at an average heart rate of 400~600 bpm and at 37°C. B-mode was used to obtain 2-dimensional real time long axis view images for strain analysis to measure EF and cardiac output. Short axis view images were captured using M-mode at the mid ventricular level and visualized by the presence of papillary muscles to measure ventricular dimension, fractional shortening, and wall thickness.

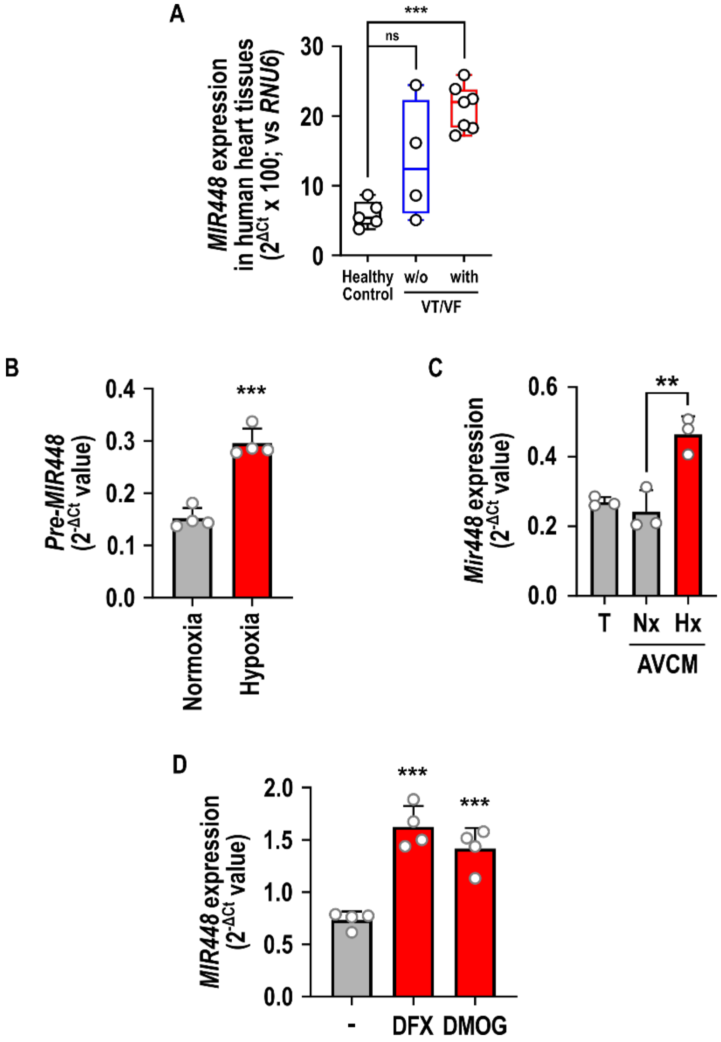

Figure. S1. Effect of hypoxic condition on the expression miR-448.

(A) Expression of miR-448 in the left ventricle of controls or human heart failure patients with or without ventricular tachycardia or ventricular fibrillation. Using our tissue bank, we identified 11 patients that had cardiomyopathy whose arrhythmia status was documented in the records. Seven patients had documented ventricular tachycardia or ventricular fibrillation. When comparing these patients to controls, there was a statistically significant increase in miR-448 in the left ventricle. miR-448 levels showed a trend of increasing as a function of arrhythmic risk.

(B) Effect of hypoxia on the precursor miR-448 level in cardiomyocytes. RL14 cells were incubated in normoxic (21% O_2) and hypoxic (2% O_2) conditions for 6 h.

(C) Effect of hypoxia on the miR-448 level in the isolated mouse adult ventricular cardiomyocytes (AVCM). AVCMs were incubated in normoxic (21% O_2; Nx) and hypoxic (2% O_2; Hx) conditions for 24 h. T: acutely isolated control hearts.

(D) Effect of hypoxia-mimetic media on the miR-448 level in cardiomyocytes. RL14 cells were stimulated with desferrioxamine (DFX) and dimethyloxalylglycine (DMOG) for 24 h.

Data are represented as the mean + standard deviation (SD) of four independent experiments. **, $P<0.01$; ***, $P<0.001$

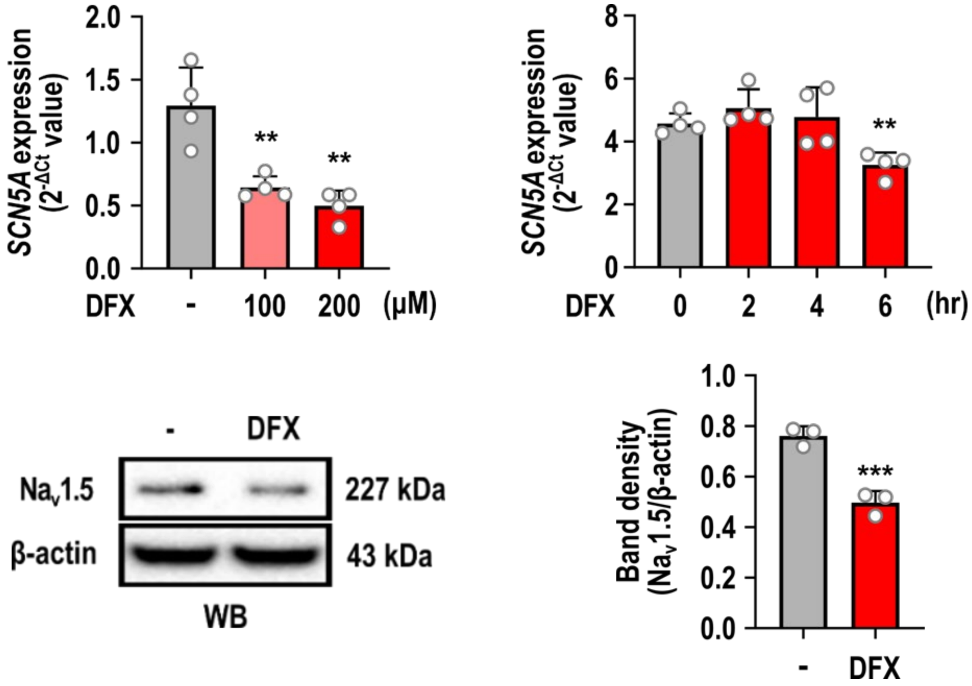

Figure. S2. Effect of DFX on the expression of *SCN5A* in RL14 human cardiomyocytes.

(Top) Effect of DFX on the *SCN5A* mRNA level in cardiomyocytes. Cells were stimulated with DFX in a dose- and time-dependent manner.

(Bottom) Effect of DFX on the protein level of *SCN5A* in cardiomyocytes. Cells were stimulated with DFX (100 μM) for 24 h.

Data are represented as the mean + standard deviation (SD) of three to four independent experiments. **, $P<0.01$; ***, $P<0.001$

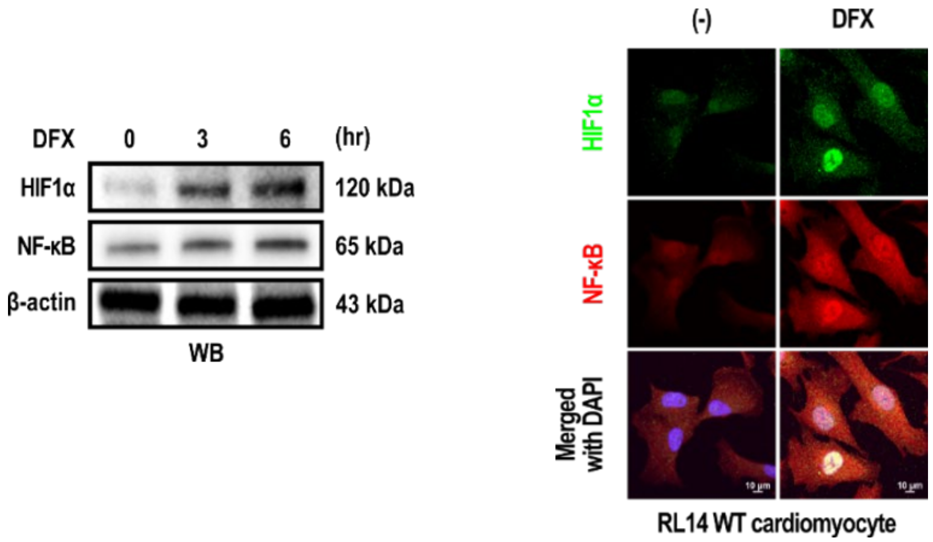

Figure. S3. Effect of DFX on the expression and translocation of HIF1α and NF-κB.

Effect of DFX on the HIF1α and NF-κB in RL14 cells. Cells were stimulated with DFX for 6 hr. The expression and nuclear translocalization of HIF1α and NF-κB was determined using western blotting and confocal microscopy.

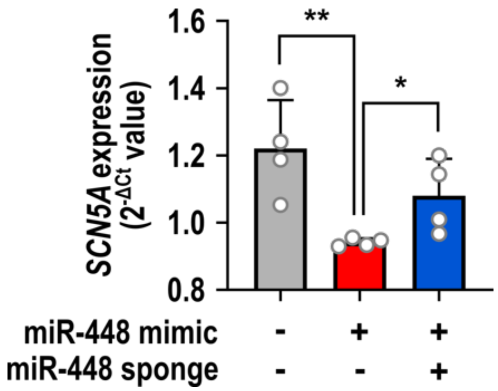

Figure. S4. Effect of miR-448 sponge on miR-448 mimic-mediated reduction of *SCN5A*.

Effect of miR-448 sponge on the expression of *SCN5A* mRNA reduced by miR-448 mimic in cardiomyocytes. Cells were transfected with miR-448 mimic in a presence or absence of miR-448 sponge.

Data are represented as the mean + standard deviation (SD) of four independent experiments. *, $P<0.05$; **, $P<0.01$

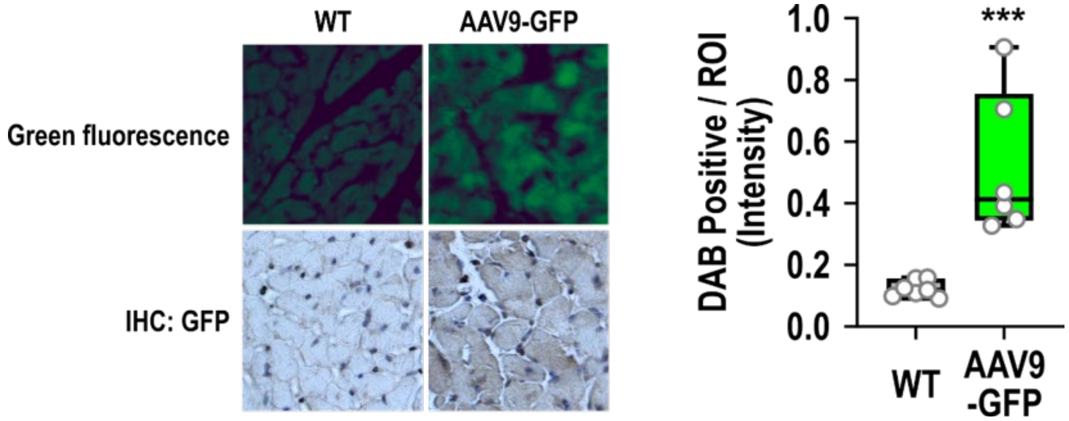

Figure. S5. GFP expression by AAV9 in mouse heart tissue.

AAV9 particles expressing GFP were injected by IV. After 2 weeks, green fluorescence was determined using fluorescence microscopy. IHC of GFP antigen done using formalin-fixed, paraffin-embedded heart tissues. Tissue sections were incubated with GFP antibody. Positively stained cells were evaluated using Image J analysis.

Data are represented as the mean ± standard deviation (SD). ***, $P<0.001$

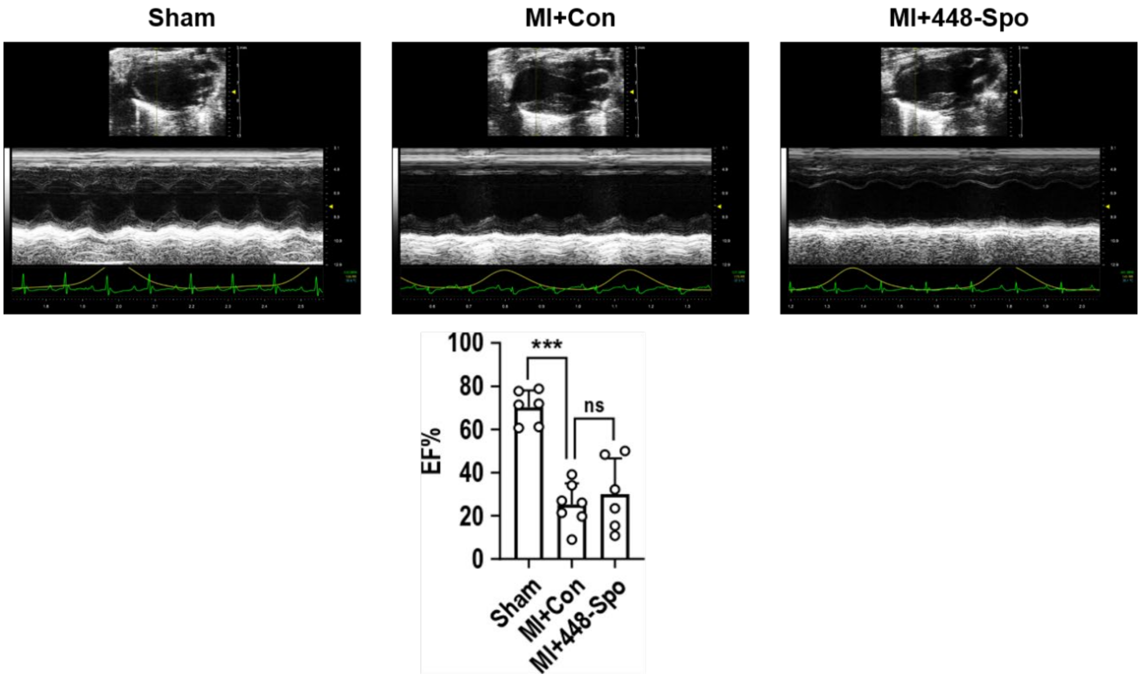

Figure. S6. Echocardiogram analysis in mice with AAV9-Con and AAV9-448-Spo.

(Top) Representative mouse M-mode echocardiography images of the mice in each group. (Bottom) Measurements of the ejection fraction (EF), n = 6–7/group.

Data are represented as the mean + standard deviation (SD). ***, *P*<0.001

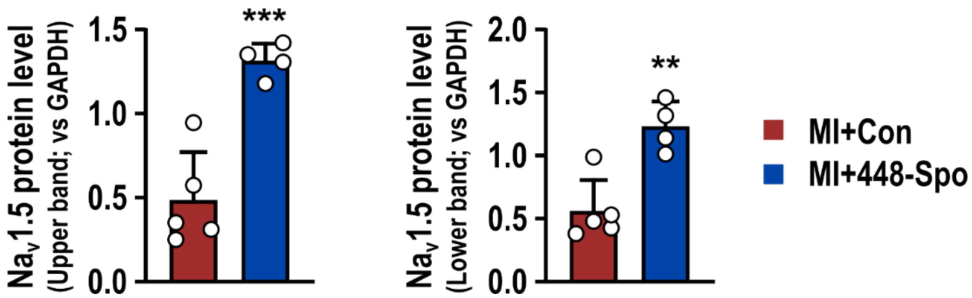

Figure. S7. Densitometric analysis of protein bands of $Na_v1.5$.

The upper or lower band was analyzed separately. Band density was measured relative to β-actin using the Image J program. Data are represented as the mean + standard deviation (SD). **, $P<0.01$; ***, $P<0.001$
